# Acute rhinosinusitis among pediatric patients with allergic rhinitis: A nationwide, population-based cohort study

**DOI:** 10.1371/journal.pone.0211547

**Published:** 2019-02-12

**Authors:** Shi-Wei Lin, Sheng-Kai Wang, Ming-Chi Lu, Chun-Lung Wang, Malcolm Koo

**Affiliations:** 1 Division of Pediatrics, Dalin Tzu Chi Hospital, Buddhist Tzu Chi Medical Foundation, Dalin, Chiayi, Taiwan; 2 Division of Allergy, Immunology and Rheumatology, Dalin Tzu Chi Hospital, Buddhist Tzu Chi Medical Foundation, Dalin, Chiayi, Taiwan; 3 School of Medicine, Tzu Chi University, Hualien City, Hualien, Taiwan; 4 Graduate Institute of Long-Term Care, Tzu Chi University of Science and Technology, Hualien City, Hualien, Taiwan; 5 Dalla Lana School of Public Health, University of Toronto, Toronto, Ontario, Canada; University of Connecticut Health Center, UNITED STATES

## Abstract

**Background:**

While chronic rhinosinusitis is a common complication of allergic rhinitis, the link between acute rhinosinusitis and allergic rhinitis is unclear. The aim of this study was to evaluate the risk of incident acute rhinosinusitis among pediatric patients with allergic rhinitis, using a nationwide, population-based health claims research database.

**Methods:**

Newly diagnosed allergic rhinitis patients aged 5–18 years were identified from the health claim records of the Longitudinal Health Insurance Database 2000 of Taiwan’s National Health Insurance Research Database. A comparison cohort was assembled by randomly selecting patients from the same database with frequency matching by sex, age group, and index year. All patients were followed until a diagnosis of acute rhinosinusitis or the end of the follow-up period. Cox proportional hazards model was used to assess the association between allergic rhinitis and acute rhinosinusitis.

**Results:**

Of the 43,588 pediatric patients included in this study, 55.4% were male and 43.9% were between the ages of 5.0–7.9 years. The risk of acute rhinosinusitis was significantly higher in pediatric patients with allergic rhinitis compared to those without the condition (adjusted hazard ratio = 3.03, 95% confidence interval = 2.89–3.18). Similar hazard ratios were observed between male and female pediatric patients.

**Conclusions:**

This secondary cohort study using a nationwide, population-based health claim data of the Taiwan’s NHIRD showed that allergic rhinitis was significantly associated with a higher risk of acute rhinosinusitis among pediatric patients.

## Introduction

Both allergic rhinitis and rhinosinusitis are widely prevalent diseases in children associating with enormous health care burden [1–3]. Although these two diseases have previously been regarded as distinct clinical entities, they are increasingly being considered as part of a spectrum of upper airway inflammatory disease [4]. There are two main types of acute rhinosinusitis: viral and bacterial. The primary symptoms associated with acute rhinosinusitis in children include cough, nasal discharge, congestion, postnasal drip, and facial pain or pressure [5]. According to the clinical practice guidelines of the American Academy of Pediatrics, acute bacterial sinusitis is defined as an infection of the paranasal sinuses lasting less than 30 days and often presenting an acute upper respiratory tract infection (URI) with persistent illness (i.e., nasal discharge or daytime cough or both) lasting more than 10 days, a worsening course, or severe onset (concurrent fever with a temperature ≥ 39°C and purulent nasal discharge for at least three consecutive days) [6]. The complications of rhinosinusitis include the local spread of disease intraorbitally or intracranially [7]. Although early antibacterial therapy can shorten the duration of illness and prevent infectious involvement of the orbit or central nervous system [8], the increasing rates of antimicrobial resistance in pediatric patients with acute rhinosinusitis has become a serious issue [9].

Over the past 50 years, an upsurge in allergic rhinitis has been observed throughout the world [10]. A survey conducted in 2002 in central Taiwan estimated that the prevalence of childhood allergic rhinitis was 27.6% [11]. Allergic rhinitis is a chronic inflammatory disease of the nasal mucosa, induced by an immunoglobulin E (IgE)-mediated reaction in allergen-sensitized individuals [12]. This mucosal inflammatory reaction could directly obstruct sinus drainage and augmented rhinosinusitis in mouse models [13]. In addition, individuals with allergic IgE-mediated rhinitis were found to have more severely impaired paranasal sinus functioning compared with nonallergic individuals during viral colds and therefore, might have a higher risk of bacterial rhinosinusitis [14]. A study comparing children with grass pollen induced rhinitis and those without inhalant allergy showed that grass pollen induced rhinitis is a negligible risk factor for acute rhinosinusitis [15].

Although it is well known that chronic rhinosinusitis is a common complication of allergic rhinitis [16], the link between acute rhinosinusitis and allergic rhinitis remains controversial. Therefore, the aim of this study was to evaluate the risk of incident acute rhinosinusitis among children with allergic rhinitis, using a nationwide, population-based health claims research database.

## Methods

### Study design and data sources

We used a population-based, retrospective cohort study design to conduct a secondary data analysis based on the Longitudinal Health Insurance Database 2000 (LHID 2000) of Taiwan’s National Health Insurance Research Database (NHIRD) [17].

The study protocol of the present study was reviewed and approved by the institutional review board of Dalin Tzu Chi Hospital, Buddhist Tzu Chi Medical Foundation, Taiwan (no. B10403028). Since the NHIRD files contain only deidentified secondary data, the need for informed consent from each individual patient was waived by the institutional review board.

### Identification of the allergic rhinitis cohort and a comparison cohort

**Fig 1:**
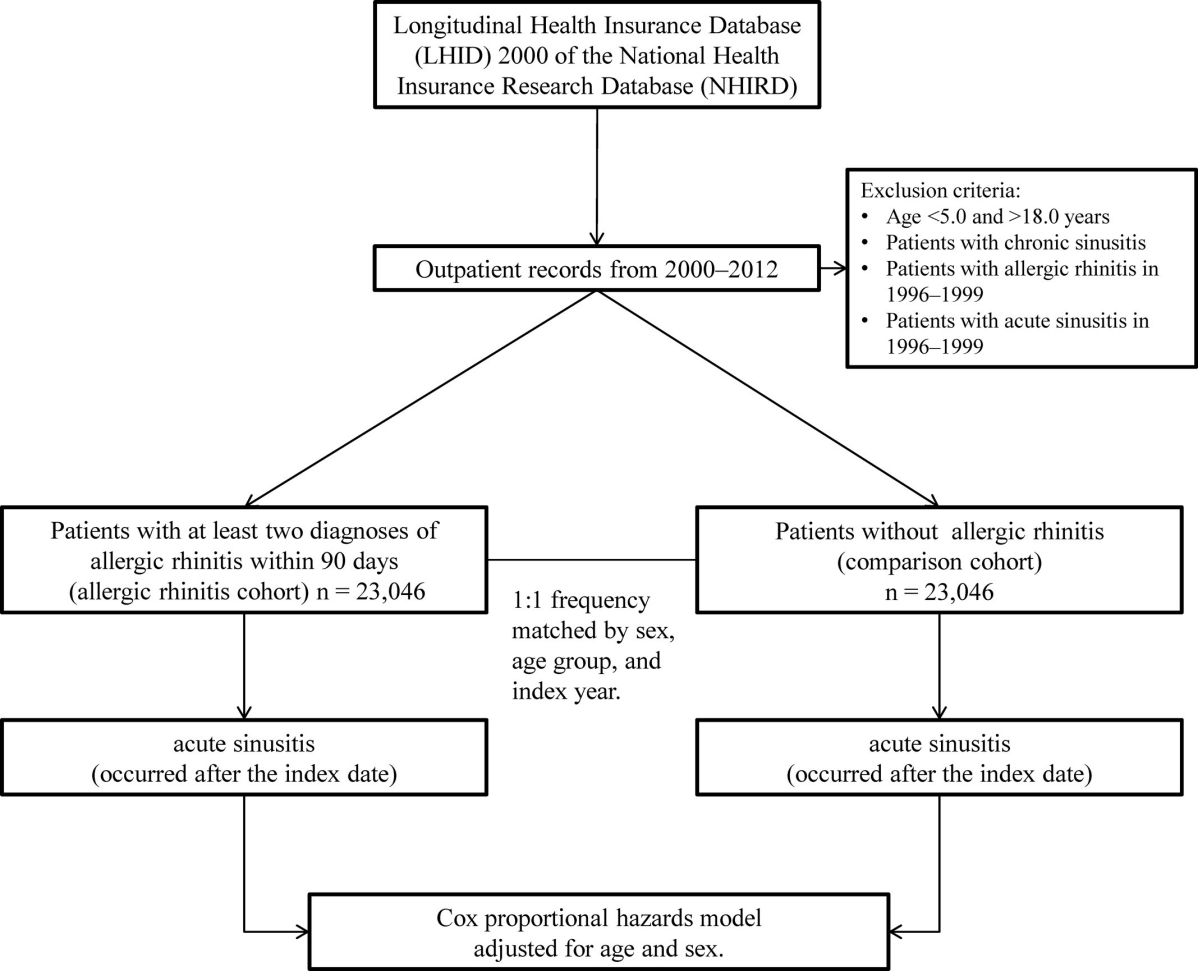
Study flowchart.

Newly diagnosed allergic rhinitis patients aged 5.0 to 18.0 years were identified from the health claim records of the LHID 2000, between January 1, 2000 to December 31, 2012, to assemble the allergic rhinitis cohort (Fig 1). To increase the validity of the diagnosis, we limited our study patients to only those with moderate to severe allergic rhinitis by defining allergic rhinitis as at least in two outpatient records with the International Classification of Diseases, Ninth Revision, Clinical Modification (ICD-9-CM) code 477.X in any diagnosis field within a period of 90 days. Patients who had been diagnosed with allergic rhinitis or acute rhinosinusitis between January 1, 1996 and December 31, 1999 were excluded from the study. In addition, patients diagnosed with chronic rhinosinusitis (ICD-9-CM code 473.X) were excluded. Patients with impairment of sinus function (ICD-9-CM code 471.X or 470.X), primary immunodeficiency (ICD-9-CM code 279.X), or failure to thrive (ICD-9-CM code 783.41) were also excluded.

A comparison cohort was assembled by randomly selecting patients from the LHID 2000 with an outpatient record between January 1, 2000 and December 31, 2012. Frequency matching by sex, age group (5.0–7.9, 8.0–10.9, 11.0–13.9, and 14.0–18.0 years), and index year was used. The same exclusion criteria used in the allergic rhinitis cohort were applied to the comparison cohort; namely, patients with allergic rhinitis or acute rhinosinusitis between January 1, 1996 and December 31, 1999 and patients with chronic rhinosinusitis were excluded from the study. Patients with impairment of sinus function, primary immunodeficiency, or failure to thrive were also excluded.

### Identification of acute rhinosinusitis

All patients were followed until a diagnosis of acute rhinosinusitis or the end of the follow-up period, which was defined as the last date of an outpatient visit for each patient. Acute rhinosinusitis was identified as a diagnosis of ICD-9-CM codes 461.0 or 461.9 that recorded in at least three outpatient visits within a period of 90 days. Any acute rhinosinusitis that occurred prior to the index date was excluded from the analysis.

### Statistical analysis

Categorical data were summarized with frequencies and percentages. Chi-square test was used to assess the differences in sex and age between the allergic rhinitis and the comparison cohorts. Kaplan-Meier survival curves were constructed and compared using log-rank test. Cox proportional hazards model was used to assess the association between allergic rhinitis and acute rhinosinusitis, with and without stratification by sex and age groups. The proportional hazards assumption was tested by adding a time-dependent covariate. Statistical significance was inferred at a two-tailed *P*-value of < 0.05. All statistical analyses were conducted using IBM SPSS Statistics for Windows, Version 24.0 (IBM Corp, Armonk, NY, USA).

## Results

**Fig 2:**
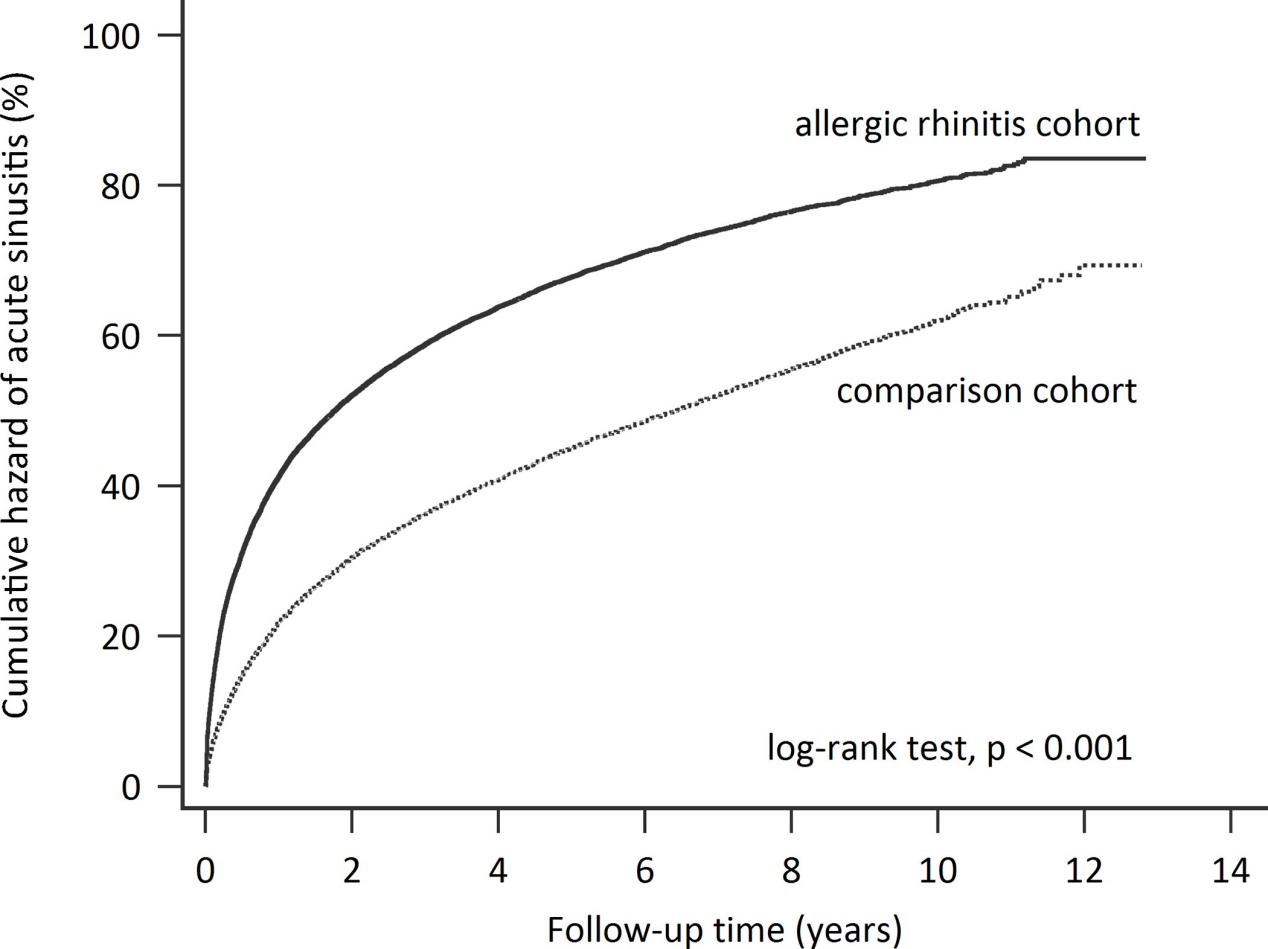
Kaplan-Meier plot of cumulative hazard over time for pediatric patients with or without allergic rhinitis.

Of the 43,588 pediatric patients included in this study, 55.4% were male and 43.9% were between the ages of 5.0–7.9 years. Since patients in the comparison cohort was assembled with frequency matching by sex and age group, no significant differences were observed between the two cohorts (Table 1). The hazard rate for acute rhinosinusitis was significantly higher for the allergic rhinitis cohort compared with that of the comparison cohort (Fig 2, log-rank test, *P* < 0.001).

**Table 1:** Demographics between pediatric patients with and without allergic rhinitis.

Variable	Total	Allergic rhinitis cohort	Comparison cohort	*P*
	N = 43588	n = 21794	n = 21794	
Sex, n (%)				> 0.999
Male	12067 (55.4)	12067 (55.4)	12067 (55.4)	
Female	19454 (44.6)	9727 (44.6)	9727 (44.6)	
Age group, years, n (%)				> 0.999
5.0–7.9	19146 (43.9)	9573 (43.9)	9573 (43.9)	
8.0–10.9	10194 (23.4)	5098 (23.3)	5098 (23.3)	
11.0–13.9	7258 (16.7)	3628 (16.7)	3628 (16.7)	
14.0–18.0	6990 (16.0)	3495 (16.0)	3495 (16.0)	

The incidence rates and hazard ratios of acute rhinosinusitis with or without stratification by sex and age group are shown in Table 2. The overall incidence rate for acute rhinosinusitis among patients with allergic rhinitis was 111.8 per 1,000 person-years compared with 33.9 per 1,000 person-years for those without allergic rhinitis. The adjusted hazard ratio (HR) was 3.03 (95% confidence interval [CI] = 2.89–3.18). Similar HRs were observed between male (HR = 3.05, 95% CI = 2.87–3.25) and female (HR = 3.01, 95% CI = 2.80–3.23) pediatric patients. The magnitude of HRs varied slightly across age groups with the highest and lowest HRs in the 14 to 18 years group (HR = 3.28, 95% CI = 2.36–4.56) and the 11 to 13.9 years group (HR = 2.64, 95% CI = 2.22–3.15), respectively.

**Table 2:** Incidence rates and hazard ratios of acute sinusitis in pediatric patients with and without allergic rhinitis, stratified by sex and age group.

	Allergic rhinitis cohort			Comparison cohort			Adjusted HR (95% CI)	*P*
	acute rhinosinusitis	person-years	IR	acute rhinosinusitis	person-years	IR		
**All**	6217	55610	111.8	2489	73327	33.9	3.03 (2.89–3.18)	< 0.001
**Stratified by sex**								
Male	3595	31054	115.8	1466	41898	35.0	3.05 (2.87–3.25)	< 0.001
Female	2622	24556	106.8	1023	31430	32.5	3.01 (2.80–3.23)	< 0.001
**Stratified by age group, years**								
5.0–7.9	4417	27814	158.8	1777	42004	42.3	3.02 (2.86–3.20)	< 0.001
8.0–10.9	1181	15017	78.6	491	18393	26.7	2.82 (2.54–3.13)	< 0.001
11.0–13.9	427	8323	51.3	177	9448	18.7	2.64 (2.22–3.15)	< 0.001
14.0–18.0	192	4456	43.1	44	3483	12.6	3.28 (2.36–4.56)	< 0.001

IR: incidence rate per 1,000 person-years; HR: hazard ratio; CI: confidence interval.

Hazard ratios obtained from Cox proportional hazards model were adjusted by age.

## Discussion

This secondary cohort study using a nationwide, population-based health claim data of the Taiwan’s NHIRD showed that allergic rhinitis was significantly associated with a higher risk of acute rhinosinusitis among pediatric patients. The incidence rate of acute rhinosinusitis in pediatric patients with allergic rhinitis was 111.8 per 1,000 person-years, which was three times over that of patients without allergic rhinitis. The incidence rate of acute rhinosinusitis was 33.9 per 1,000 person-years, which is within the range of 15–40 reported in the literature [18].

The pathogenesis of rhinosinusitis involves three key factors: sinus ostia obstruction, ciliary dysfunction, and thickening of sinus secretions [19]. We have previously reported that children over six years of age with acute rhinosinusitis combined with allergic rhinitis had significantly lower nasal peak expiratory flow rate (nPEFR) values compared with nonatopic children [20]. Our hypothesis is that nasal mucosa inflammation induced by allergic rhinitis could cause sinus ostia obstruction. Viral upper respiratory tract infections could then lead to ciliary dysfunction and thickening of sinus secretions. Therefore, patients with underlying disease of allergic rhinitis could be at an increased risk of developing acute rhinosinusitis when attacked by viral upper respiratory tract infections [21].

Allergic rhinitis is frequently associated with complications and comorbid conditions, including allergic conjunctivitis, chronic rhinosinusitis, and asthma [22]. Previous research indicated that antibiotic treatment alone was unable to resolve symptoms of acute rhinosinusitis in children with allergy [23]. Taken together with the increased risk of acute rhinosinusitis among pediatric patients with allergic rhinitis observed in this study, more aggressive management of allergic rhinitis in pediatric patients should be considered.

To our knowledge, this study is the first to report an increased rate of acute rhinosinusitis in pediatric patients with allergic rhinitis using a population-based, retrospective cohort study design. Nevertheless, a few limitations should be taken into account when interpreting the findings. First, there is a lack of information on the severity of acute rhinosinusitis, which is a limitation common to all studies based on analysis of the NHIRD. Second, we could not confirm that all the acute rhinosinusitis in our study was acute bacterial sinusitis. However, acute bacterial sinusitis typically follows a viral rhinosinusitis, and clinical diagnosis of acute bacterial sinusitis is generally based on history [24]. Third, detection bias could occur when patients in the allergic rhinitis cohort had a higher frequency of medical visits compared to those in the comparison cohort. Nevertheless, given the highly affordable out-of-pocket medical copayment under the Taiwan’s National Health Insurance scheme, it is unlikely to be the cause of the observed increase in risk of acute rhinosinusitis among patients with allergic rhinitis.

In summary, this secondary cohort study using a nationwide, population-based health claim database showed that pediatric patients with allergic rhinitis was significantly associated with three times increased risk of developing acute rhinosinusitis. Further studies evaluating the effectiveness of a more aggressive management of allergic rhinitis in lowering the risk of acute rhinosinusitis among pediatric patients may be warranted.
